# National Trends in Gender-Affirming Surgical Procedures: A Google Trends Analysis

**DOI:** 10.7759/cureus.25906

**Published:** 2022-06-13

**Authors:** Emily Merrick, Joshua P Weissman, Mona Ascha, Sumanas W Jordan, Marco Ellis

**Affiliations:** 1 Plastic and Reconstructive Surgery, Northwestern University Feinberg School of Medicine, Chicago, USA

**Keywords:** top surgery, gender affirming surgery, google trends, non-binary, transgender

## Abstract

Background: There has been a significant increase in the volume of gender-affirming surgical (GAS) procedures over the past decade. The objective of this paper is to use online search data from Google Trends (GT) to describe national search trends for GAS procedures.

Methods: GT was queried for search terms relating to GAS from January 2004 to February 2021. The 19 selected keywords covered a broad range of GAS topics. United States (US) search interest was collected as relative search volumes (RSVs) and then analyzed by geographic region. The number of plastic surgery providers offering GAS and academic surgery centers was collected from the World Professional Association for Transgender Health (WPATH) and Trans-health.com. RSVs were analyzed by metro area to determine the relationship between search demand and personal income. State Medicaid policies for transgender health services were also collected.

Results: All search terms demonstrated a positive increase in RSVs over time except “sex reassignment surgery” and “penectomy”. The Mountain/Pacific and East South Central/West South Central had the greatest search volume for GAS and most providers offering care. The East South Central/West South Central region​​ ranked last for providers offering care, despite the relatively high search interest. This region also had no states with explicit Medicaid policies covering gender-affirming care. Metro areas in the top five for RSV but bottom quartile for per capita personal income were identified.

Conclusions: Online search interest for GAS-related terms has increased. Search interest for GAS has regional variation and did not show a specific pattern with provider availability.

## Introduction

Approximately 1.4 million transgender and non-binary (TGNB) adults and 150,000 TGNB adolescents and young adults live in the United States (US) [1]. Gender-affirming surgery (GAS) has become increasingly performed over the past five years for this growing patient population [2]. GAS has been shown to improve quality of life among TGNB patients experiencing gender dysphoria [3-6]. Many patients consult internet forums and social media for information regarding GAS [7,8]. El-Hadi et al. found that websites targeted at TGNB patients were the primary source of information for GAS. Furthermore, the authors found that the majority of TGNB individuals had difficulty finding a physician and reported having a lack of access to information [3]. 

Search engines such as Google can provide valuable information about healthcare-related search trends. The largest keyword search engine, Google Trends (GT), is a free, accessible tool that allows individuals to analyze geographic and temporal trends as relative search volumes (RSVs) for search terms [9]. GT is gaining popularity in healthcare-related research [10-14]. As has been done in prior studies, these RSVs can be used as a proxy for both search demand and interest [14-19]. Prior plastic surgery research has utilized GT to predict public interest in various surgical procedures, understand demand for marketing purposes, perceive celebrity influences on procedure interest, and conduct geographical analysis of provider demand [14-19]. A recent study utilizing GT reported increased searches for GAS-related search terms globally [19]. 

GT has not been utilized to analyze US interest in GAS. The purpose of this study is to describe US trends in internet searches for GAS-related keywords by region and over time. We hypothesize that there will be discrepancies across various national regions between GT search demand for GAS such that areas with more surgical providers and gender centers will generate higher search interest. 

## Materials and methods

Data source

GT was used to assess search volumes and trends over time [9,20]. GT evaluates the interest of a specific search term and generates an indicator known as an RSV, which is a score that calculates the relative popularity of a term as a proportion of all Google search terms for a specific geographic region or time frame [9,20]. GT analyses can be customized by search term, geographic location, time period, category (e.g. “Arts and Entertainment”, “Books and Literature”), and type of Google search (e.g. “web search”, “image search”, “news search”). This method provides anonymous, open-source data that controls population size and internet usage. Each data point is divided by the total number of searches of the geography and time point it represents. The resultant numbers are scaled on a range of 0 to 100 based on a topic’s proportion to all searches on all topics within that region or time [9]. A score of 100 represents the geographic area or time period with the greatest interest for that search term. All other geographic areas and times are assigned numbers that quantify interest relative to the maximum. For example, if area or time X had a search volume of 550 searches and area or time Y had an RSV of 495 searches, GT would record area or time X as having an RSV of 100 (550/550) and area or time Y would have an RSV of 90 (495/550).

Data acquisition

GT search parameters were set to Geographic Location: “United States,” Time Period: “1/1/2004-2/18/2021,” Category: “All Categories,” and Type of Search: “Web Search” to capture all US queries for the designated terms. Specific dates were used to ensure the replicability of the study. The terms used in this analysis were among the core procedure types defined by the World Professional Association for Transgender Health (WPATH) [21]. The aim was to capture a broad scope of demand for GAS. Researcher and clinical consensus were used for final term selection as determined by the two senior authors who regularly perform GAS. Of note, the authors acknowledge that terminology such as male-to-female (“MTF”), “FTM”, and “gender confirmation surgery” may be considered outdated and/or stigmatizing to some. These terms were included in order to reflect and analyze language that shifts within a wide time frame. The search terms included in this study can be found in Table 1. “Male to female/female to male surgery” was counted as one search term as GT does not consider word order and generates the same data for both searches. Terms that did not generate any RSV values from GT were excluded from the analysis. These terms were as follows: “peritoneal flap vaginoplasty”, “Adam’s apple reduction”, “facial masculinization”, “MTF vaginoplasty”, “transmasculine bottom surgery”, and “transfeminine bottom surgery”. Surgical terms that were not specific to gender-affirming procedures such as “vaginoplasty”, “breast augmentation”, and “mastectomy” were excluded. All terms were individually queried via GT and the RSVs were determined for each search term from January 1, 2004 to February 18, 2021.

**Table 1:** Search terms used for analysis Terms were selected through the World Professional Association for Transgender Health and clinical consensus between the two senior authors who frequently perform gender affirming procedures. The authors acknowledge that some terms are outdated and stigmatizing but were utilized to reflect the changing language of this wide time frame.

Search Terms
Transgender Surgery
Top Surgery
Bottom Surgery
Upper Surgery
Lower Surgery
Sex Reassignment Surgery
Gender Reassignment Surgery
Vulvoplasty
MTF Bottom Surgery
FTM Bottom Surgery
FTM Phalloplasty
Penectomy
Male to Female Surgery / Female to Male Surgery
Facial Feminization Surgery
Tracheal Shave
Penile Skin Inversion
Metoidioplasty
Gender Affirming Surgery
Gender Confirming Surgery

Temporal data

Microsoft Excel (Redmond, WA) was used to conduct a trend line analysis for each search term’s RSV. 

Regional data

**Figure 1:**
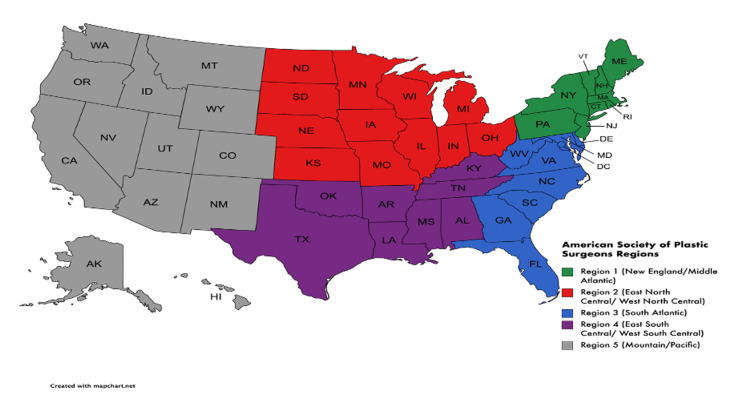
Regions defined according to the America Society of Plastic Surgeons, 2019 Plastic Surgery Statistics Report

RSV values were recorded by state for each of the 19 search terms. States were categorized according to regions designated by the American Society of Plastic Surgeons (ASPS) Annual Reports [2]. Figure 1 displays the five regions used in the analysis. The top 10 RSVs were recorded for all search terms. The total number of times each region appeared in the top ten RSVs was recorded to compare regional interest in GAS.

To determine the specific region in which each search term was most popular, the top 10 RSVs were examined. The region composed the largest percentage of RSVs for each search term’s top 10 RSVs was deemed to have the greatest regional interest for that term. For search terms where there was insufficient data to report on 10 states, the RSV for all available states was reported. In the event that multiple regions made up equal percentages (e.g., five RSVs were from Region 1 and five RSVs were from Region 2), the region with the larger average RSV for that search term was determined to have the greatest interest. 

Metropolitan area based on income data

A metropolitan analysis of RSV for each of our search terms was also conducted for all of our search terms. GT search parameters were set to Geographic Location: “United States,” Time Period: “1/1/2004-2/18/2021,” Categories: “All categories,” and Type of Search: “Web Search.” For each of our search terms, we analyzed any metro area that was in the top five for RSV but bottom quartile according to the US Bureau of Economic Analysis [22]. Metro areas were then recorded to determine demand trends in low-income areas where care may be less accessible.

Providers and academic medical centers offering GAS data

To quantify the number of plastic surgeon providers in the US offering GAS as of February 2021, the World Professional Association of Transgender Health’s (WPATH) website was utilized. The website lists plastic surgery providers in each state, and the search was subsequently filtered for these providers [21]. The number of providers listed on this website for each state were determined and the number of providers in each region were calculated. The Trans-Health website was queried for the number of academic medical centers with TGNB surgery programs [23]. Additional centers were manually added if they were known to have TGNB programs but were not listed on the website. All 154 websites of US medical schools were evaluated for access to a dedicated TGNB surgical program or dedicated GAS section, and fifteen academic medical centers were added to the original list. The number of centers were counted for each state and region. 

Medicaid coverage data

As of February 2021, the states that have an explicit policy stating that TGNB health-related services are covered under Medicaid were determined [24]. We also examined which states had a policy excluding coverage and which states did not have a specific stance. We then calculated the number of states within each region that had each of the aforementioned policies. This was done to determine whether there are associations between a state’s Medicaid status for GAS and its overall search demand for GAS-specific terms. 

## Results

Trends in interest in the United States from 2004 to 2021

Overall, national trends in search terms for gender affirming surgery revealed “transgender surgery,” “gender reassignment surgery,” “gender affirming surgery,” “gender confirming surgery” “top surgery,” “bottom surgery,” “upper surgery,” “lower surgery,” “male to female/female to male surgery,” “MTF bottom surgery,” “FTM bottom surgery,” “facial feminization surgery,” “metoidioplasty,” “FTM phalloplasty,” “tracheal shave,” “vulvoplasty,”, and “penile skin inversion.” all showed positive increases in RSV from 2004 to 2021. The two terms that showed a decrease in RSV overtime were “sex reassignment surgery” and “penectomy.” 

Interest in GAS by region

**Figure 2:**
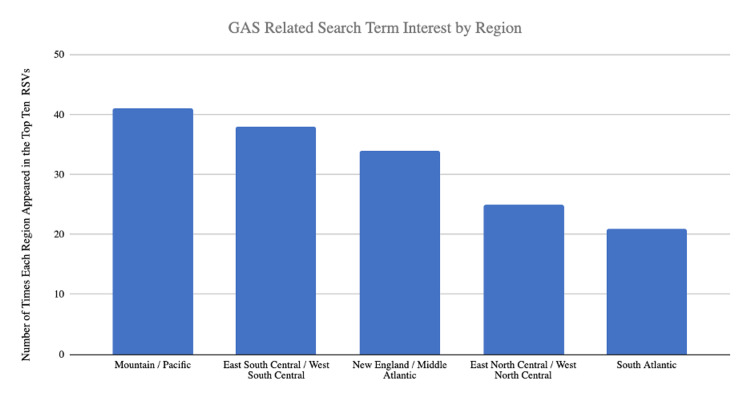
Gender-affirming surgical (GAS) related search term interest by region For all 19 search terms, the states with the top ten relative search volumes were listed. The number of times each state was included in the top 10 was noted and added into one of the five regions described by the America Society of Plastic Surgeons.

Based on GT data for all selected search term to identify interest by state and region, 41/159 (25.8%) of the top ten RSV values fell into Region 5 (Mountain/Pacific), 38/159 (23.4%) RSVs fell into Region 4 (East South Central/West South Central), 34/159 (21.4%) of RSVs fell into Region 1 (New England/Middle Atlantic), 25/159 (15.7%) fell into Region 2 (East North Central/West North Central), and 21/159 (13.2%) RSVs fell into Region 3 (South Atlantic) (Figure 2). 

Based on RSV data for all nineteen search terms, Region 5 (Mountain/Pacific) had the greatest interest for the search terms: “sex reassignment surgery,” “gender reassignment surgery,” “tracheal shave,” “penectomy,” “facial feminization surgery,” “metoidioplasty,” and “penile skin inversion.” Region 4 (East South Central/West South Central) had the greatest interest for the search terms “bottom surgery,” “male to female/female to male surgery,” “vulvoplasty,” “lower surgery.” Region 1 (New England/Middle Atlantic) had the greatest interest for “transgender surgery,” “top surgery,” “FTM phalloplasty.” Region 3 (South Atlantic) had the greatest search interest for “MTF bottom surgery,” “FTM bottom surgery,” and “upper surgery.” Region 2 (East North Central/West North Central) had the greatest interest for the search terms “gender affirming surgery” and “gender confirming surgery.”

Metropolitan data based on income

Metro areas in the top five for RSV but bottom quartile for per capita personal income included: Richmond, Virginia, Las vegas, Nevada, Spartansburg, South Carolina, Grand rapids-Kalamazoo-Battle Creek, MI, Jacksonville, Florida, Columbus, Ohio, Hartford, Connecticut, and Bowling Green Kentucky.

Providers and academic medical centers offering GAS data

**Figure 3:**
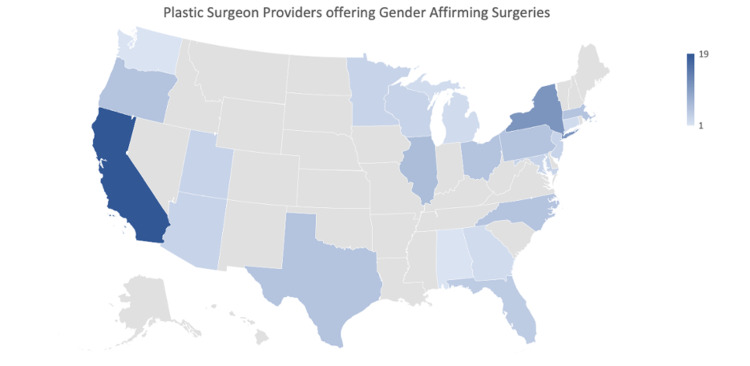
Plastic surgeon providers offering gender affirming surgeries Plastic surgeon providers across the United States as listed on the World Professional Association for Transgender Health (WPATH) website.

The WPATH website provided 101 plastic surgery providers across the US. A national distribution of these providers can be seen in Figure 3. Region 5 (Mountain/Pacific) has the most providers with 33% of the total. Region 1 (New England/Middle Atlantic) has 26%, Region 2 (East North Central/West North Central) has 19%, Region 3 (South Atlantic) has 14% of the providers. Region 4 (East South Central/West South Central) has the fewest number of providers with nine percent.

**Figure 4:**
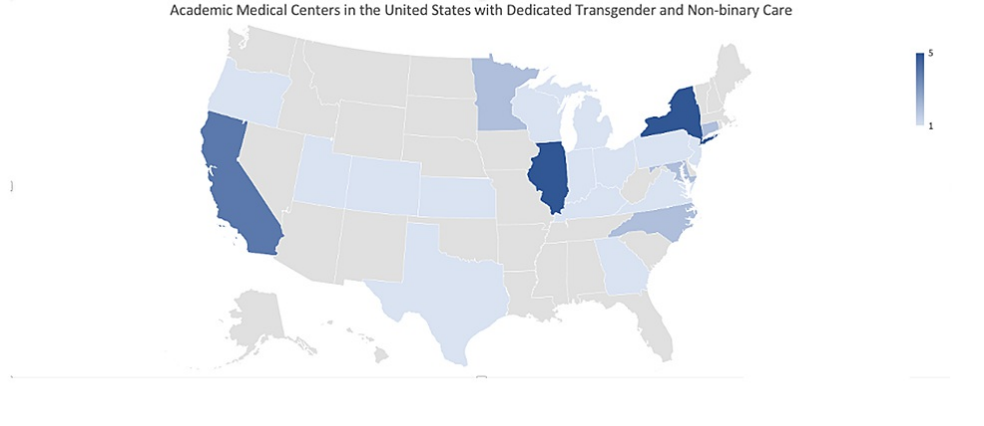
Academic medical centers in the United States with dedicated transgender and non-binary care States with academic medical centers with a dedicated transgender and non-binary surgery program or faculty dedicated to providing this type of care - World Professional Association for Transgender Health (WPATH) website.

A total of 46 academic medical centers with TGNB surgery programs or dedicated TGNB surgery sections on their websites were found. A national distribution of these institutions can be seen in Figure 4. Region 1 (New England/Middle Atlantic) had the most institutions with 14 (30%). Region 2 (East North Central/West North Central) followed with 12 institutions (26%). Regions 3 (South Atlantic) and 5 (Mountain/Pacific) had nine (20%) and eight (17%) institutions, respectively. Region 4 (East South Central/West South Central) had the fewest number of institutions with three (7%).

Medicaid coverage data

**Figure 5:**
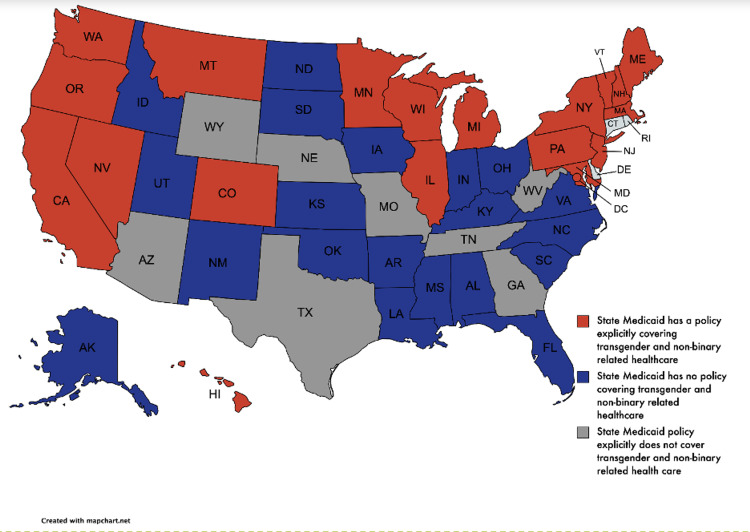
Distribution of Medicaid policies regarding coverage of transgender and non-binary care across the United States

Of the 22 states and Washington D.C. in which Medicaid programs cover gender affirming care, Region 1 (New England/Middle Atlantic) has the most states with this policy (nine states). Region 5 (Mountain/Pacific) follows with seven states, Region 2 (East North Central/West North Central) has four states, Region 3 (South Atlantic) has three states. Region 4 (East South Central/West South Central) has no states with this explicit policy. Figure 5 illustrates a national distribution of state policy.

## Discussion

We used GT to describe US search trends for various GAS procedures according to geographic region. As past studies have done, we aimed to use GT search data as a proxy for national interest in GAS [14,16-19]. Given the greater number of individuals identifying as TGNB, GAS is becoming an increasingly important component of health care in the US [25-27]. Prior studies have highlighted the rapid increase in demand for GAS [25]. We found that national search interest related to GAS has increased over time while demonstrating regional variation.

Our results highlight growing US search interest in GAS, as 17 of 19 included search terms had positive increases in RSVs from 2004 to 2021. The only terms that decreased in popularity were “sex reassignment surgery” and “penectomy.” This may be due to a shift in language used by TGNB individuals [4-7,25-27]. 

TGNB patients report that the lack of knowledgeable TGNB providers is a large barrier to care [27]. Using GT to identify areas that lack available providers may be a helpful step in addressing this barrier. Our GT analysis demonstrates that search interest for GAS does not align with areas containing providers or gender centers. This highlights a geographic barrier to accessing care. Our GT regional analysis shows that Region 5 (Mountain/Pacific) and Region 4 (East South Central/West South Central) have higher search demand volume relative to the other three regions. Despite generating relatively high search interest, Region 4 (East and West South Central) ranked last for both the number of providers and academic medical centers offering care. GT can be used to identify high interest, low availability areas where increasing providers may want to be prioritized.

Each region had certain search terms that were more widely used. For example, Region 5 (Mountain/Pacific) had the highest demand for terms such as “tracheal shave”, “penectomy”, and “facial feminization surgery” whereas Region 4 (East South Central/West South Central) had the highest demand for “vulvoplasty”. Variation in search interest for specific terms may indicate potentially popular procedures in certain regions and/or regional differences in language. Providers in these areas can use this knowledge to include popular searches in their internet presence or increase awareness about other available procedures that patients might appreciate but not be exposed to in their region.

Additionally, certain states (Wyoming, Wisconsin, Virginia, Delaware, South Dakota, and Hawaii) did not have top 10 search volumes for any of the selected terms. Of the states that did not appear in the top 10 for RSV volumes, only Wisconsin and Virginia had available providers to perform GAS. These can be targeted as areas where increasing awareness of potential procedures may be of significant value to TGNB individuals. GT can be used to track increased interest, and providers can be recruited to practice in areas of increasing interest. 

We identified eight low-income metro areas with significant search interest for GAS procedures. To ensure equitable access to care, providers can be incentivized to practice in these areas. Our results demonstrate that Region 4 (East South Central/West South Central) had the lowest rates of Medicaid coverage and the fewest providers. Increasing coverage may be another means to draw providers to certain areas. Ultimately, regional variations of demand for gender-affirming care are complex and are often the result of local and state legislation, Medicaid coverage, and culture [28].

There are several limitations to this study. Search volume values can be skewed by population size. A state with a larger population may have a higher absolute search volume but a lower proportion of total search results compared to a state with a smaller population. This limitation is minimized as numbers are taken in the context of RSVs and there are no comparisons made between absolute search volumes. In addition, only search terms with sufficient interest to generate an RSV value were included. The authors also recognize that not every term relating to GAS may have been included in this analysis. Patients may also be searching terms not specifically analyzed in this study or search terms in another language. Additionally, this study is limited in not providing information or outcomes for those actually undergoing GAS: not differentiating between the number of insured versus uninsured patients undergoing GAS, complication rates, or the types of surgeries people are getting. This offers the future-direction of questionnaire-based studies to elucidate this information. The GT algorithm generates RSV values based on a random sample of Google searches, meaning that reproducibility of our exact search is not guaranteed. It is also important to acknowledge that not all patients have internet access. Our sample only represents patients who can search online. Nevertheless, the 2018 United States Census shows that 92% of households in the US own a computer and 85% had an internet subscription [29]. ​​ Although Google has the largest search engine market share (93%), the results of this study are only representative of the demographic that uses the Google search engine users and those who use the internet for healthcare-related information [30]. 

## Conclusions

This paper reports on the utility of GT in relation to transgender health and GAS. We describe national trends in online search interest based on region, city, and metropolitan area. Our results highlight a strong, growing national interest in GAS. Search interest for GAS has regional variation and did not show a specific pattern with provider availability. By improving our understanding of temporal and geographical search interest, we can identify areas where patients may seek GAS. 
